# Development of a Nomogram Combining Clinical Risk Factors and Dual-Energy Spectral CT Parameters for the Preoperative Prediction of Lymph Node Metastasis in Patients With Colorectal Cancer

**DOI:** 10.3389/fonc.2021.689176

**Published:** 2021-09-22

**Authors:** Yuntai Cao, Jing Zhang, Haihua Bao, Guojin Zhang, Xiaohong Yan, Zhan Wang, Jialiang Ren, Yanjun Chai, Zhiyong Zhao, Junlin Zhou

**Affiliations:** ^1^ Department of Radiology, Affiliated Hospital of Qinghai University, Xining, China; ^2^ Second Clinical School, Lanzhou University, Lanzhou, China; ^3^ Department of Radiology, Lanzhou University Second Hospital, Lanzhou, China; ^4^ Key Laboratory of Medical Imaging of Gansu Province, Lanzhou, China; ^5^ Gansu International Scientific and Technological Cooperation Base of Medical Imaging Artificial Intelligence, Lanzhou, China; ^6^ Department of Radiology, The Fifth Affiliated Hospital of Zunyi Medical University, Zhuhai, China; ^7^ Department of Radiology, Sichuan Provincial People’s Hospital, Chengdu, China; ^8^ Department of Critical Medicine, Affiliated Hospital of Qinghai University, Xining, China; ^9^ Department of Hepatopancreatobiliary Surgery, Affiliated Hospital of Qinghai University, Xining, China; ^10^ Department of Pharmaceuticals Diagnosis, General Electrics (GE) Healthcare, Beijing, China

**Keywords:** tomography, X-ray computed, colorectal cancer, lymph node metastasis, nomogram

## Abstract

**Objective:**

This study aimed to develop a dual-energy spectral computed tomography (DESCT) nomogram that incorporated both clinical factors and DESCT parameters for individual preoperative prediction of lymph node metastasis (LNM) in patients with colorectal cancer (CRC).

**Material and Methods:**

We retrospectively reviewed 167 pathologically confirmed patients with CRC who underwent enhanced DESCT preoperatively, and these patients were categorized into training (*n* = 117) and validation cohorts (*n* = 50). The monochromatic CT value, iodine concentration value (IC), and effective atomic number (Eff-Z) of the primary tumors were measured independently in the arterial phase (AP) and venous phase (VP) by two radiologists. DESCT parameters together with clinical factors were input into the prediction model for predicting LNM in patients with CRC. Logistic regression analyses were performed to screen for significant predictors of LNM, and these predictors were presented as an easy-to-use nomogram. The receiver operating characteristic curve and decision curve analysis (DCA) were used to evaluate the clinical usefulness of the nomogram.

**Results:**

The logistic regression analysis showed that carcinoembryonic antigen, carbohydrate antigen 199, pericolorectal fat invasion, ICAP, ICVP, and Eff-ZVP were independent predictors in the predictive model. Based on these predictors, a quantitative nomogram was developed to predict individual LNM probability. The area under the curve (AUC) values of the nomogram were 0.876 in the training cohort and 0.852 in the validation cohort, respectively. DCA showed that our nomogram has outstanding clinical utility.

**Conclusions:**

This study presents a clinical nomogram that incorporates clinical factors and DESCT parameters and can potentially be used as a clinical tool for individual preoperative prediction of LNM in patients with CRC.

## Introduction

According to the latest global cancer statistics, colorectal cancer (CRC) is ranked among the top three cancers in terms of both prevalence and mortality, and its incidence is increasing (1). Accurate preoperative evaluation of lymph node metastasis (LNM) is critical to making a precise treatment plan and evaluating patient prognosis (2, 3). Although histopathological features such as tumor differentiation and lymphatic invasion are closely related to LNM, these features are only available postoperatively and provide limited clinical guidance (4). Accurate assessment of LNM preoperatively provides valuable information for patients with CRC to choose the optimal treatment plan, thereby improving their prognosis.

Despite clinical advances, LNM evaluation remains a challenging issue for radiologists. Non-invasive radiological modalities, such as CT, magnetic resonance imaging, and endoluminal ultrasonography, have been widely utilized in the evaluation of LNM in clinical practice. However, these imaging methods cannot accurately evaluate LNM using criteria such as short-axis diameter, signal heterogeneity, shape, and boundary (2, 5, 6). Therefore, developing more sensitive diagnostic tools for the preoperative prediction of LNM in patients with CRC patients is imperative.

The application of dual-energy spectral computed tomography (DESCT) is considered a milestone in the history of CT diagnosis. DESCT expands the single parameter scanning mode of conventional CT, providing multiple quantitative parameters, such as monochromatic images at energy levels of 40~140 keV, material decomposition images (such as iodine-based or water-based decomposition images), and effective atomic number (Eff-Z) images. Based on this advantage, DESCT has been widely applied in clinical practice for such uses as CRC grading, malignant lymph node (LN) identification, neoadjuvant treatment therapy response evaluation, and microsatellite instability status evaluation (7–10). Previous studies have evaluated the value of DESCT in distinguishing metastatic and non-metastatic LNs preoperatively in CRC (8, 11); however, the ability of DESCT for predicting LNM in primary CRC has not been evaluated. Several nomograms have been developed to predict LNM in CRC (4, 6, 12). Huang et al. developed a nomogram to predict LNM of CRC based on CT radiomics features; the nomogram showed good predictive performance in both training and validation cohorts. Li et al. proposed a clinical-radiomics nomogram with a combination of clinical risk factors and radiomics features for preoperative prediction of LNM in patients with CRC; the nomogram had moderate discrimination performance. Zhou et al. established a nomogram for LNM prediction in patients with rectal cancer based on clinical factors; the AUC of the nomogram was 0.743 in the training cohort and 0.777 in the validation cohort. However, it is not clear whether incorporating clinical risk factors and DESCT parameters in a nomogram would improve its predictive ability for LNM in patients with CRC. Therefore, the purpose of our study was to develop a clinical–DESCT nomogram that incorporated both clinical factors and DESCT parameters for individual preoperative prediction of the risk of LNM in CRC.

## Materials and Methods

### Patients

**Figure 1:**
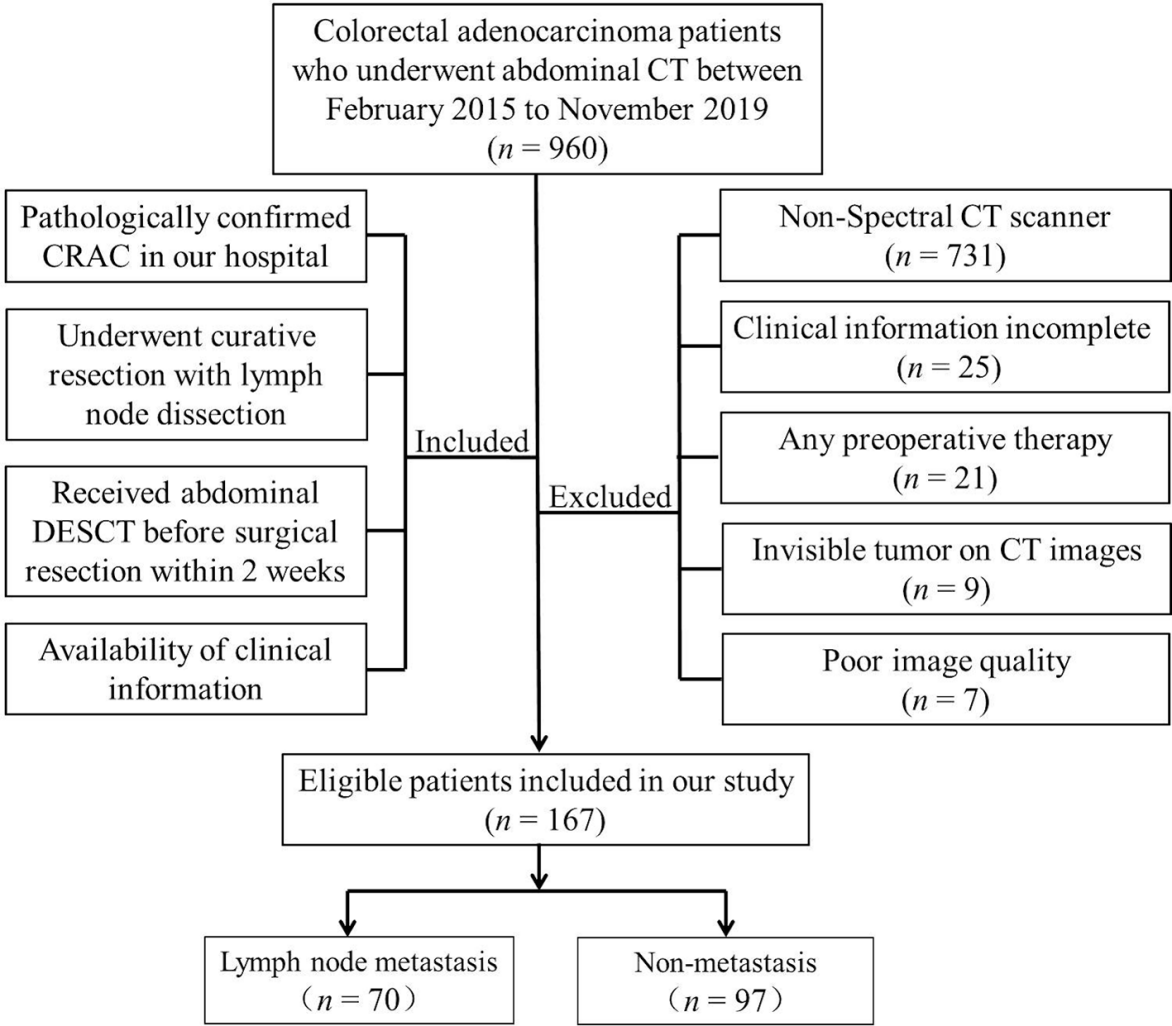
A flow diagram of patient recruitment, including inclusion and exclusion criteria.

This study was approved by the Institutional Review Board of Lanzhou University Second Hospital Medical Ethics Committee, and the requirement for informed consent was waived. We retrospectively evaluated all patients seen at our hospital between February 2015 and November 2019; eligible patients were those with pathologically confirmed CRC who underwent curative resection with LN dissection and who had received abdominal enhanced DESCT imaging before surgery. A flow diagram of the recruitment pathway, including inclusion and exclusion criteria, is shown in **Figure 1**. A total of 167 patients were identified and included in our study (85 with colon cancer; 82 with rectal cancer), and these patients were categorized into the training and validation cohorts. Clinical data and preoperative tumor serologic data were collected by reviewing the medical records of patients. Data collected were age, sex, tumor location, preoperative carbohydrate antigen 199 (CA19-9), preoperative carbohydrate antigen 125 (CA125), and preoperative carcinoembryonic antigen (CEA) levels. The status of LNM was evaluated by trained pathologists. The LN-positive group was characterized by the presence of one or more metastatic LNs, while the LN-negative group was defined by normal healthy LNs.

### DESCT Imaging

All patients underwent bowel preparation before the examination. Contrast-enhanced abdominal dual-energy CT scans were performed using a Discovery CT750 HD system (GE Healthcare, Waukesha, WI, USA) in the supine position. The energy spectrum CT scanning protocol was as follows: fast tube voltage switching between 80 and 140 kVp; tube current, 350 mAs; rotation time, 0.75 s; pitch, 0.984:1; and reconstructed layer thickness, 1.25 mm. Patients were intravenously injected with iodixanol (1 ml/kg) at an injection rate of 3.5–4.5 ml/s using a high-pressure dual-cylinder injector. Arterial phase (AP) and venous phase (VP) imaging were performed 25–30 and 60–70 s after the administration of contrast agent, respectively.

### Image Postprocessing and Analysis

**Figure 2:**
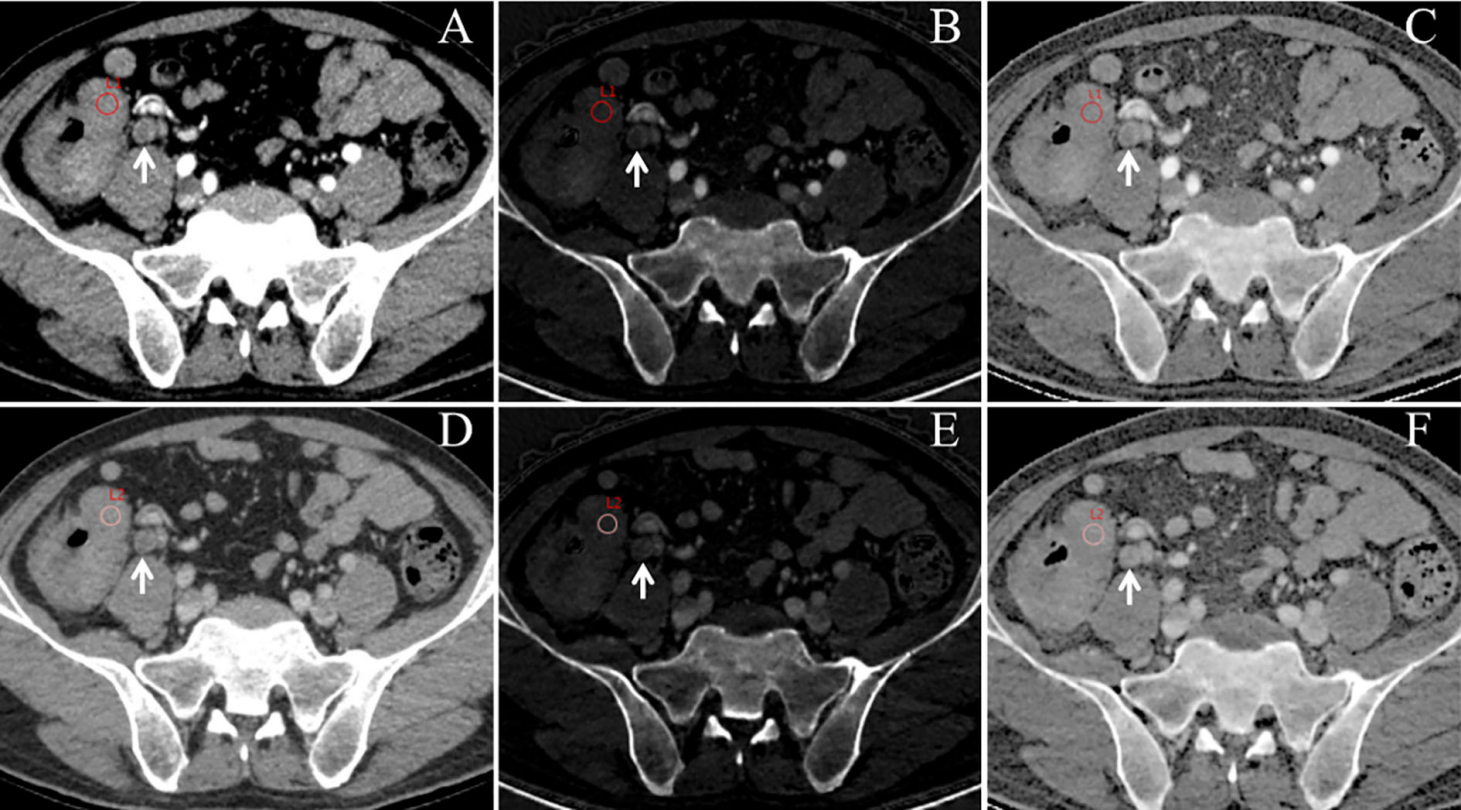
An example of dual-energy spectral computed tomography (DESCT) images with regions of interest (ROIs) for evaluating quantitative measurements in a 63-year-old man with ascending colon cancer that was pathologically confirmed to have lymph node metastasis (LNM). ROIs were placed in the arterial phase **(A)** and the venous phase **(D)** of the 70-keV monochromatic images. Concurrently, ROIs were copied to the arterial phase **(B)** and the venous phase **(E)** of iodine-based material decomposition images and the arterial phase **(C)** and the venous phase **(F)** of the effective atomic number images. Local lymphadenopathy is presented in front of the right psoas major (white arrow) at both the arterial phase and venous phase **(A–F)**.

**Figure 3:**
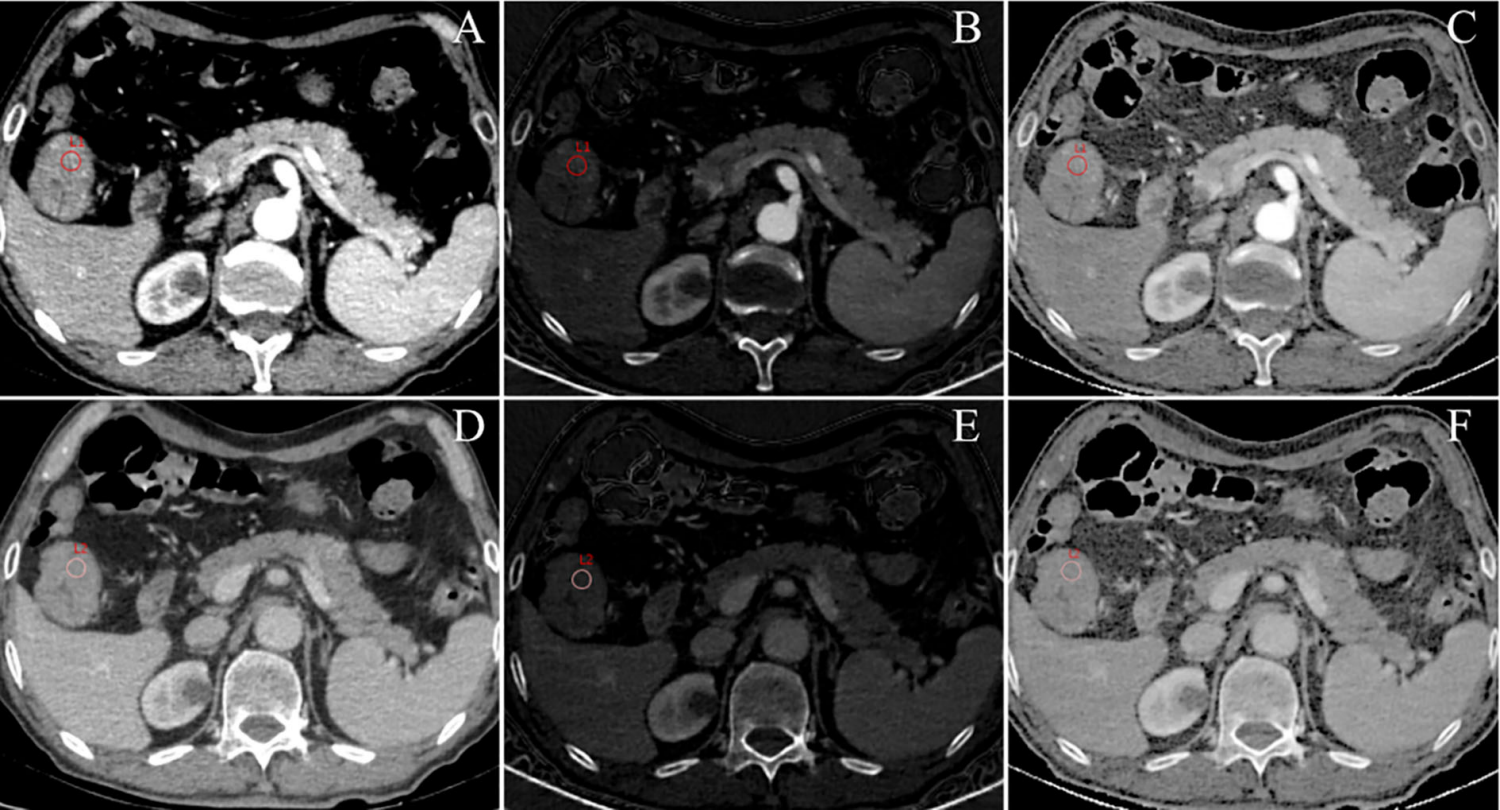
An example of DESCT images with ROIs for evaluating quantitative measurements in a 75-year-old man with ascending colon cancer that was pathologically confirmed to have non-metastatic lymph nodes. ROIs were placed in the arterial phase **(A)** and the venous phase **(D)** of the 70-keV monochromatic images. At the same time, ROIs were copied to the arterial phase **(B)** and the venous phase **(E)** of iodine-based material decomposition images and the arterial phase **(C)** and venous phase **(F)** of the effective atomic number images.

Raw CT imaging data were transferred to a GE ADW 4.6 workstation (GE Healthcare, Milwaukee, WI, USA). Gemstone Spectral Imaging (GSI) viewer software was used to quantitatively evaluate virtual monochrome images with a default of 70 keV, iodine-based decomposition images, and Eff-Z images. Two radiologists with more than 5 years of experience in gastrointestinal radiology performed the image analysis. Both radiologists were blinded to the clinical and pathological data of the patients. The maximum tumor thickness was defined as the maximum diameter perpendicular to the long axis on the cross-sectional image. Pericolorectal fat invasion (PFI) was defined as the extension of the primary tumor beyond the muscularis propria and its invasion of the pericolorectal fat. Clinical tumor (cT) stage was evaluated according to the eighth edition of the American Joint Committee on Cancer Staging system (13). Two radiologists independently drew circular regions of interest (ROIs) at the maximum slice of the tumor with an average area of 97.27 mm^2^ on 70 keV monochromatic images in the AP and VP. Previous studies have shown that 70 keV monochromatic images may provide an optimal trade-off between sensitivity and specificity for abdominal lesion analysis (14). ROIs were placed on solid areas to avoid vascular, necrotic, and cystic changes as much as possible. Tumor monochromatic CT values, iodine concentration (IC) values, and Eff-Z values were generated using the GSI viewer software package. To minimize bias, all measurements were performed three times and the average of the three values was taken as the final value. Examples of DESCT images with ROIs for evaluating quantitative measurements in two patients with CRC with and without LNM are shown in **Figures 2** and **3**, respectively.

### Statistical Analysis

All statistical analyses were performed using the R statistical software package (version 3.6.3; http://www.Rproject.org). Student’s *t*-test or Mann–Whitney *U* tests were used to compare continuous variables between the LN-positive and the LN-negative groups. Chi-square tests or Fisher’s exact tests were used to compare categorical variables. A two-sided *p*-value <0.05 was considered statistically significant. The intraclass correlation coefficient (ICC) was used to calculate the consistency of measurements between the two radiologists. The statistically significant features in the univariate analysis were included in a multivariate logistic regression analysis. Backward stepwise selection was applied, in which the stopping rule was the likelihood ratio test with Akaike’s information criterion. A multivariable logistic regression analysis was performed to select parameters with predictive significance for LNM. A quantitative and easy-to-use nomogram was built based on the final regression coefficient and designed to predict the individual probability of LNM. Receiver operating characteristic curve (ROC) analysis was used to evaluate the diagnostic capabilities of the nomogram, including calculation of the AUC value and 95% confidence interval (CI). The accuracy, sensitivity, specificity, positive predictive value (PPV), and negative predictive value (NPV) were also calculated. To verify the clinical usefulness of the nomogram, we quantified the net benefit at different threshold probabilities in the data set using decision curve analysis (DCA).

## Results

### Interobserver Agreement

Substantial interobserver agreement between the two radiologists was noted for all measurements. The ICCs for the 70-keV monochromatic CT values in the AP (CTAP), CT values in the VP (CTVP), IC values in the AP iodine concentration in the arterial phase (ICAP), IC values in the VP iodine concentration in the venous phase (ICVP), maximum tumor thickness, Eff-Z in the AP (Eff-ZAP), Eff-Z in the VP (Eff-ZVP), PFI, and cT stage were 0.912, 0.905, 0.928, 0.938, 0.922, 0.894, 0.887, 0.915, and 0.846, respectively.

### Associations Between Clinical Variables and LNM

A total of 167 patients with CRC were included in the final analysis. The LN-positive group comprised 70 patients, with an average age of 60.07 ± 12.86 years, and 54.3% of the patients were males. The LN-negative group comprised 97 patients, with an average age of 59.83 ± 12.15 years, and 60.8% of the patients were males. We used stratified sampling to categorize the study cohort into a training cohort (*n* = 117) and a validation cohort (*n* = 50). The training cohort was used for model building, while the validation cohort was used for internal validation of the model. Patient and tumor characteristics in the training and validation cohorts are listed in **Table 1**.

**Table 1:** Clinical characteristics and DESCT parameters of CRC patients [mean ± SD or no. (%)].

Characteristics	Training cohort (*n* = 117)			Validation cohort (*n* = 50)		
	LN metastasis (−)	LN metastasis (+)	*p*-value	LN metastasis (−)	LN metastasis (+)	*p*-value
Age (years)	60.04 ± 12.09	60.41 ± 11.58	0.960	59.31 ± 14.47	59.29 ± 15.75	0.922
Gender Female	28 (41.2)	19 (38.8)	0.794	10 (34.5)	13 (61.9)	0.055
Male	40 (58.8)	30 (61.2)		19 (65.5)	8 (38.1)	
Tumor location Left	46 (67.6)	32 (65.3)	0.791	19 (65.5)	16 (76.2)	0.416
Right	22 (32.4)	17 (34.7)		10 (34.5)	5 (23.8)	
CEA level Normal	44 (64.7)	23 (46.9)	0.055	20 (69.0)	4 (19.0)	<0.001
Abnormal	24 (35.3)	26 (53.1)		9 (31.0)	17 (81.0)	
CA125 level Normal	64 (94.1)	44 (89.8)	0.387	25 (86.2)	19 (90.5)	0.647
Abnormal	4 (5.9)	5 (10.2)		4 (13.8)	2 (9.5)	
CA19-9 level Normal	62 (91.2)	32 (65.3)	<0.001	24 (82.8)	11 (52.4)	0.021
Abnormal	6 (8.8)	17 (34.7)		5 (17.2)	10 (47.6)	
Maximum diameter (cm)	20.75 ± 10.37	20.36 ± 7.09	0.564	21.69 ± 11.52	22.93 ± 9.51	0.438
cT stage T1–2	20 (29.4)	10 (20.4)	0.271	7 (24.1)	2 (9.5)	0.184
T3–4	48 (70.6)	39 (79.6)		22 (75.9)	19 (90.5)	
Gross tumor pattern Non-polypoid	60 (88.2)	39 (79.6)	0.201	26 (89.7)	14 (66.7)	0.045
Polypoid	8 (11.8)	10 (20.4)		3 (10.3)	7 (33.3)	
Pericolorectal fat invasion No	40 (58.8)	7 (14.3)	<0.001	13 (44.8)	2 (9.5)	0.007
Yes	28 (41.2)	42 (85.7)		16 (55.2)	19 (90.5)	
ICAP	17.52 ± 4.53	20.65 ± 3.19	<0.001	17.14 ± 4.28	20.43 ± 3.75	0.002
ICVP	15.15 ± 2.25	17.36 ± 2.77	<0.001	15.35 ± 2.50	17.14 ± 2.85	0.024
Eff_ZAP	8.67 ± 0.30	8.79 ± 0.36	0.107	8.64 ± 0.28	8.82 ± 0.34	0.101
Eff_ZVP	8.54 ± 0.33	8.71 ± 0.30	0.004	8.54 ± 0.36	8.64 ± 0.38	0.132
CTAP (HU)	81.40 ± 10.63	80.98 ± 12.84	0.600	81.98 ± 11.87	80.67 ± 11.69	0.534
CTVP (HU)	74.00 ± 9.23	73.86 ± 9.48	0.494	74.39 ± 8.07	77.81 ± 9.94	0.400

LN, lymph node.

In the training cohort, the LN-positive group showed higher CA19-9 level and PFI compared with the LN-negative group (all *p*-values <0.05; **Table 1**). The values for the DESCT parameters ICAP, ICVP, and Eff-ZVP were also significantly higher for the LN-positive group compared with those for the LN-negative group (all *p*-values <0.05; **Table 1**). There were no significant differences in other clinical factors and tumor DESCT parameters between the LN-positive group and the LN-negative group in the training cohort (all *p*-values >0.05).

### Prediction Model Analysis

**Figure 4:**
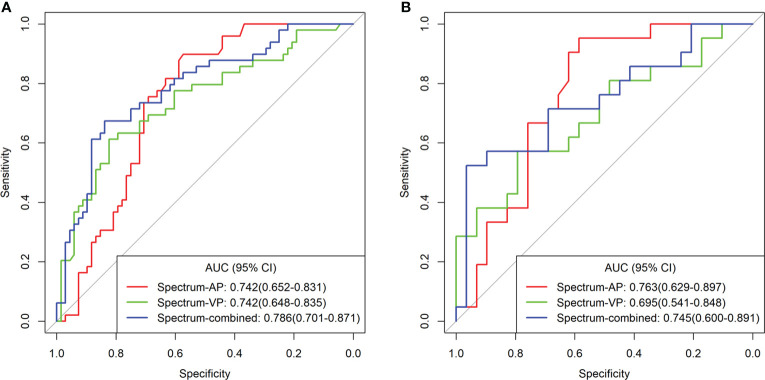
Comparison of the different spectrum models for the identification of LNM in patients with colorectal cancer in the training cohort **(A)** and validation cohort **(B)**.

Logistic regression analysis showed that CEA level, CA19-9 level, PFI, ICAP, ICVP, and Eff-ZVP were independent predictors in the predictive model. Based on these predictors, the following five predictive models were established to predict the individual probability of LNM: one clinical model, three DESCT models, and one combined clinical–DESCT model (**Table 2**). **Figure 4** shows the classification performance of the spectrum-AP model, spectrum-VP model, and spectrum-combined model. The AUC values of the spectrum-AP model, spectrum-VP model, and spectrum-combined model were 0.742 (95% CI, 0.652–0.831), 0.742 (95% CI, 0.648–0.835), and 0.786 (95% CI, 0.701–0.871), respectively, in the training cohort, and they were 0.763 (95% CI, 0.629–0.897), 0.695 (95% CI, 0.541–0.848), and 0.745 (95% CI, 0.600–0.891), respectively, in the validation cohort.

**Table 2:** Predictive performance of different models in training and validation cohorts.

Models	Training cohort						Validation cohort					
	AUC	Accuracy	Sensitivity	Specificity	PPV	NPV	AUC	Accuracy	Sensitivity	Specificity	PPV	NPV
Clinicoradiological	0.769 (0.689–0.848)	0.701 (0.609–0.782)	0.857 (0.682–0.943)	0.588 (0.308–0.698)	0.600 (0.544–0.623)	0.851 (0.750–0.871)	0.727 (0.598–0.857)	0.640 (0.492–0.771)	0.905 (0.692–1.000)	0.448 (0.252–0.640)	0.543 (0.476–0.568)	0.867 (0.785–0.903)
Spectrum-AP	0.742 (0.652–0.831)	0.709 (0.618–0.790)	0.898 (0.735–0.959)	0.574 (0.326–0.706)	0.603 (0.554–0.618)	0.886 (0.816–0.906)	0.763 (0.629–0.897)	0.740 (0.597–0.854)	0.905 (0.429–1.000)	0.621 (0.310–0.793)	0.633 (0.450–0.656)	0.900 (0.818–0.920)
Spectrum-VP	0.742 (0.648–0.835)	0.735 (0.645–0.812)	0.612 (0.347–0.735)	0.824 (0.574–0.912)	0.714 (0.586–0.750)	0.747 (0.672–0.765)	0.695 (0.541–0.848)	0.600 (0.452–0.736)	0.571 (0.381–0.905)	0.621 (0.447–0.931)	0.522 (0.421–0.633)	0.667 (0.590–0.750)
Spectrum-combined	0.786 (0.701–0.871)	0.769 (0.682–0.842)	0.673 (0.306–0.796)	0.838 (0.603–0.927)	0.750 (0.577–0.780)	0.781 (0.719–0.798)	0.745 (0.600–0.891)	0.680 (0.533–0.805)	0.571 (0.381–0.811)	0.759 (0.483–1.000)	0.632 (0.533–0.709)	0.710 (0.609–0.763)
Nomogram	0.876 (0.815–0.936)	0.812 (0.729–0.878)	0.755 (0.489–0.878)	0.853 (0.691–0.956)	0.787 (0.706–0.811)	0.829 (0.797–0.844)	0.852 (0.748–0.956)	0.760 (0.618–0.869)	0.762 (0.429–1.000)	0.759 (0.552–0.931)	0.696 (0.562–0.750)	0.815 (0.762–0.844)

Clinicoradiological, fusion of clinical risks and radiological features; Spectrum-combined, fusion of spectrum-AP and spectrum-VP; Nomogram, fusion of clinical risks, radiological features, and spectrum parameters.

AUC, area under the curve; CI, confidence interval; PPV, positive predictive value; NPV, negative predictive value; AP, arterial phase, VP venous phase.

**Figure 5:**
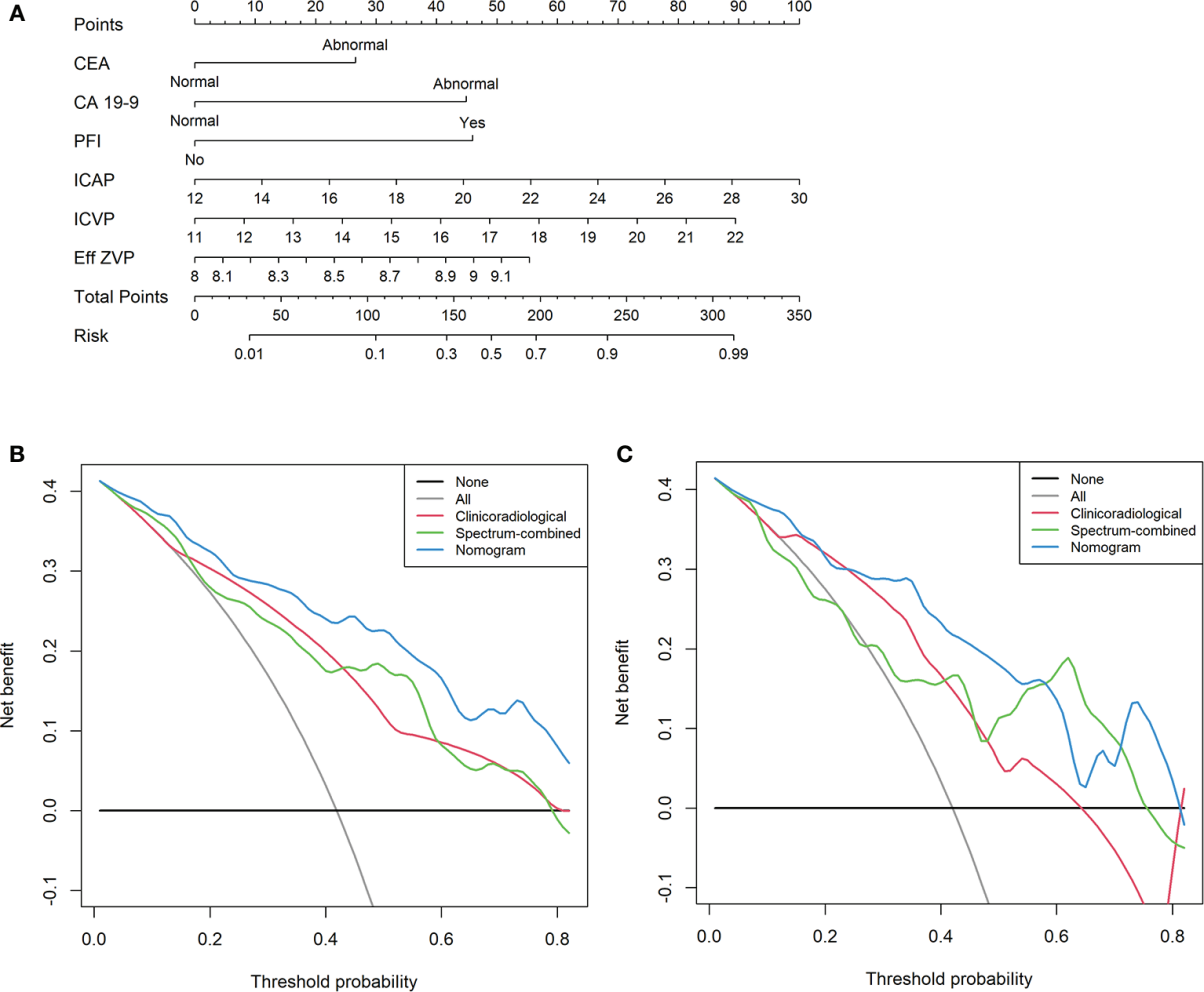
A multiparametric clinical–DESCT nomogram for predicting the probability of LNM in CRC patients **(A)**. Decision curve analysis (DCA) of the clinical–DESCT nomogram, clinicoradiological model, and spectrum-combined model in the training cohort **(B)** and validation cohort **(C)**.

**Figure 6:**
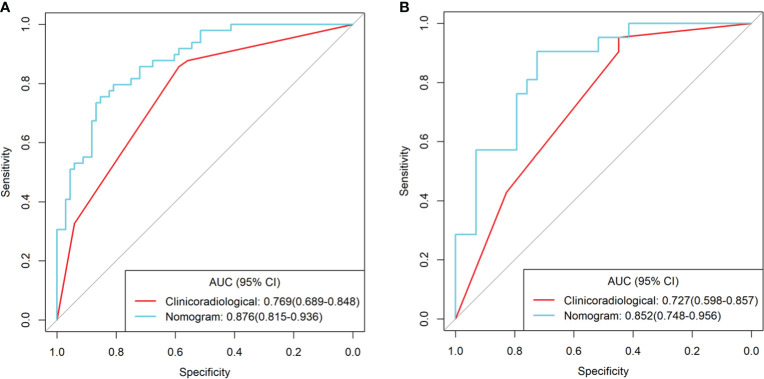
ROC curves of the clinical–DESCT nomogram, clinicoradiological model, and spectrum-combined model for preoperative prediction of LNM in patients with colorectal cancer in the training cohort **(A)** and validation cohort **(B)**.

We developed a clinical–DESCT model that combined two clinical features: one morphological image feature and three DESCT parameters; the model was presented as a quantitative nomogram (**Figure 5A**). We found that the nomogram had higher prediction contributions than the clinicoradiological model or spectrum models (**Table 2** and **Figure 6**). The AUC, accuracy, sensitivity, specificity, PPV, and NPV were 0.876, 0.812, 0.755, 0.853, 0.787, and 0.829, respectively, in the training cohort, while they were 0.852, 0.760, 0.762, 0.759, 0.696, and 0.815, respectively, in the validation cohort. The DCA for the clinical–DESCT nomogram, clinicoradiological model, and spectrum-combined model is presented in **Figures 5B, C**. The clinical–DESCT nomogram was more clinically useful, i.e., it predicted the risk of LNM more accurately than the single clinicoradiological model and single spectrum-combined model in the training and validation cohorts. The DCA demonstrated that the nomogram had the highest clinical benefit when the threshold probability was 22.6%–58.3% in both training and validation cohorts. The best threshold obtained from ROC was 0.502, which falls within this interval, indicating that the nomogram had the highest performance and clinical benefit among other models in this study.

## Discussion

To the best of our knowledge, this is the first study to construct a clinical–DESCT model that combined clinical risk factors and DESCT features of primary lesions for preoperative prediction of LNM in patients with CRC. Herein, we first screened the preoperatively available risk factors for independent predictors using multivariate logistic regression analysis. Then, we incorporated these clinical and DESCT risk factors into an easy-to-use nomogram to facilitate their use in clinical practice. The nomogram showed higher predictive accuracy than other clinicoradiological and spectrum models for LNM both in the training cohort and validation cohort. The DCA showed that the actual benefits of the model were excellent. The clinical application of this nomogram facilitates the individual preoperative prediction of LNM and thus helps develop more reasonable and effective therapeutic strategies.

Several previous studies identified metastatic and non-metastatic LNs in CRC using DESCT. Liu et al. (11) used energy spectrum CT to identify metastatic and non-metastatic LNs in patients with rectal cancer; they found that when combining NIC in the VP with the short-axis diameter, the overall AUC was 0.819. Al-Najami et al. (8) evaluated the value of dual-energy CT in identifying metastatic LNs in rectal cancer; several DESCT parameters showed moderate diagnostic accuracy for LNM. However, these studies only focused on the LNs, ignoring the features of the primary tumor. Histopathological features of primary CRCs, such as tumor differentiation and lymphatic invasion, are crucial for the development of LNM (15). In the present study, we extracted the DESCT parameters from the primary tumor and found that there were significant differences in the DESCT parameters between the LN-positive and LN-negative groups in both the arterial and venous phases. DESCT parameters of arterial and venous phases showed moderate predictive accuracy for LNM.

In our study, we found that preoperative CEA level, CA19-9 level, PFI, ICAP, ICVP, and Eff-ZVP were independent risk factors for LNM. In terms of clinical features, high CEA and CA19-9 levels are an important risk factor for LNM in patients with CRC, as reported in previous studies (4, 6). Elevated CEA and CA19-9 levels indicate increased tumor aggressiveness and metastasis (6). In addition, the LN-positive group displayed a higher incidence of PFI than did the LN-negative group in our study. This finding shows that CRC with LNM has a more aggressive behavior, involving LNs as well as peripheral fat around the primary tumor. Preoperative PFI status was a qualitative feature that could be easily obtained *via* CT imaging. Our study found that PFI status was an independent risk factor for LNM. Surprisingly, we found that DESCT parameters such as ICAP, ICVP, and Eff-ZVP were also independent predictors for LNM. These findings suggest that quantitative imaging provides significant variables for the construction of predictive nomograms. Therefore, we combined both clinical risk factors and DESCT parameters into the nomogram for preoperative prediction of LNM in patients with CRC. We found that this nomogram had higher predictive AUC and greater net benefits than single clinicoradiological and single spectrum-combined models. Hence, the clinical–DESCT combined model may be the most promising approach to predict LNM in patients with CRC.

In this study, in terms of DESCT features, ICAP and ICVP were significantly higher in the LN-positive group than in the LN-negative group, and both ICAP and ICVP were independent predictors for LNM in the multivariate analysis. ICs can evaluate the degree of tumor vascularization as this measure can quantitatively reflect the deposition of iodine in the tissue (10). IC in the AP reflects the functional capillary density, while IC in the VP reflects iodine equilibrium in the blood vessels (16). Therefore, the ICs in the AP and the VP reflect the dynamic distribution of iodine in the tumor tissue. Tumor angiogenesis is closely related to tumor growth, progression, and metastasis (17, 18). Heterogeneity in tumor angiogenesis leads to differences in the biological behavior of tumors, such as LNM (19). High angiogenesis intensity is closely related to aggressive histopathological features, such as LNM, in CRC (20–22). In the present study, we found that the LN-positive group had significantly higher IC values than the LN-negative group, suggesting that the primary tumors of patients with LNM had a greater blood supply.

The Eff-Z is an indication of tissue density, i.e., the higher the density, the higher the Eff-Z (23). In the present study, we found that Eff-ZVP was statistically higher in the LN-positive group than in the LN-negative group in the training cohort. The higher Eff-Z values observed in the LN-positive group in our study might indicate a relatively compact cell structure within the tumor; this finding is consistent with previous reports (24). In a recent investigation of ADC values in early and advanced colon tumors, a significantly lower mean ADC value was observed in advanced compared with early tumors, with an optimal discrimination cutoff value of 1.179 × 10^−3^ mm^2^/s. The ADC value has a negative correlation with the tissue cell density, i.e., the higher the density of the tissue, the lower the ADC value.

In the training cohort, we found that the predictive accuracy of LNM in the spectrum-AP model was similar to that in the spectrum-VP model and increased to 0.769 when the two phases were combined. However, double DESCT scans increase the radiation dose more than single DESCT scans. Further research aimed at developing a technique to reduce the radiation dose is necessary; for example, the application of multimodel iterative reconstruction technology can reduce the radiation dose effectively and improve the image quality (25).

This study has several limitations. First, owing to the retrospective nature of the study, there was an inevitable selection bias that should be addressed in future prospective and external validation studies. Second, the number of patients in this study was small, and the results should be further validated with larger samples. Finally, we did not perform histopathologic–radiologic, one-to-one matching of LNs, as this study focused on the primary tumor and the peripheral LNs were not evaluated using DESCT.

In conclusion, we constructed a clinical–DESCT nomogram that incorporates both clinical risk factors and DESCT parameters, presenting a non-invasive and highly useful predictive tool for individual preoperative prediction of LNM in patients with CRC.

## Data Availability Statement

The raw data supporting the conclusions of this article will be made available by the authors, without undue reservation.

## Ethics Statement

The studies involving human participants were reviewed and approved by the Medical Ethics Committee of Lanzhou University Second Hospital. The ethics committee waived the requirement of written informed consent for participation.

## Author Contributions

Conception and design: HB, JuZ, and YuC. Collection and assembly of data: YuC and XY. Development of the methodology: JR. All authors contributed to the article and approved the submitted version.

## Funding

This work was supported by the National Natural Science Foundation of China (grant number 82071872, 81772006), Open Fund Project of Key Laboratory of Medical Imaging of Gansu Province (GSYX202009), Science and Technology Project of Qinghai Province (No. 2017-SF-158), and Qinghai Provincial Key Clinical Specialty Construction Project.

## Conflict of Interest

The authors declare that the research was conducted in the absence of any commercial or financial relationships that could be construed as a potential conflict of interest.

## Publisher’s Note

All claims expressed in this article are solely those of the authors and do not necessarily represent those of their affiliated organizations, or those of the publisher, the editors and the reviewers. Any product that may be evaluated in this article, or claim that may be made by its manufacturer, is not guaranteed or endorsed by the publisher.

## Abbreviations

AP, arterial phase; CA125, carbohydrate antigen 125; CA19-9, carbohydrate antigen 199; CEA, carcinoembryonic antigen; CRC, colorectal cancer; CI, confidence interval; DCA, decision curve analysis; DESCT, dual-energy spectral CT; Eff-Z, effective atomic number; IC, iodine concentration; ICC, intraclass correlation coefficients; LNM, lymph node metastasis; PFI, pericolorectal fat invasion; ROC, receiver operating characteristic; ROI, region of interest; TGP, tumor gross pattern; VP, venous phase
